# Associations of cord blood meta-inflammation and vitamin D with neurodevelopmental delay: A prospective birth cohort study in China

**DOI:** 10.3389/fimmu.2022.1078340

**Published:** 2023-01-04

**Authors:** Peng Wang, Lin Wu, Wan-jun Yin, Rui-xue Tao, Ying Zhang, Pei-pei Li, Xiao-min Jiang, Zi-yu Shao, Peng Zhu

**Affiliations:** ^1^Department of Maternal, Child & Adolescent Health, School of Public Health, Anhui Medical University, Hefei, China; ^2^MOE Key Laboratory of Population Health Across Life Cycle, Hefei, China; ^3^NHC Key Laboratory of Study on Abnormal Gametes and Reproductive Tract, Hefei, China; ^4^Anhui Provincial Key Laboratory of Population Health and Aristogenics, Hefei, China; ^5^Anhui Provincial Institute of Translational Medicine, Hefei, Anhui, China; ^6^Department of Obstetrics and Gynecology, the First People’s Hospital of Hefei City, Hefei, China; ^7^Department of Obstetrics and Gynecology, the First Affiliated Hospital of Anhui Medical University, Hefei, China; ^8^Maternal and Child Health, and Family Planning Service Center, Hefei, China; ^9^Department of Obstetrics and Gynecology, Anhui Women and Child Health Care Hospital, Hefei, China

**Keywords:** inflammation, immune activation, fetal hyperinsulinemia, vitamin D, neurodevelopment

## Abstract

**Aim:**

To estimate the associations of cord meta-inflammatory markers with neurodevelopment, including the potential impact of cord blood vitamin D levels.

**Method:**

The prospective cohort study comprised 7198 participants based on the Maternal & Infants Health in Hefei study. Cord blood C-peptide, high-sensitive C-reactive protein (hsCRP), high-density lipoprotein-cholesterol, low-density lipoprotein-cholesterol, total cholesterol, triglycerides and 25(OH)D levels were measured. The Gesell Developmental Schedules were used to assess neurodevelopmental outcomes in offspring.

**Results:**

After adjusting potential confounders, per quartile increase in cord blood 25(OH)D concentrations was associated with a decreased risk of neurodevelopmental delay [hazard ratios (HR) 0.65 (95% CI 0.57, 0.74)]. Conversely, significant positive associations with cord blood serum C-peptide levels above the 90th percentile [HR 2.38 (95% CI 1.81, 3.13)] and higher levels of cord hsCRP (per quartile increase) [HR 1.18 (95% CI 1.01, 1.37)] with neurodevelopmental delay were observed. These associations could vary by quartiles of cord blood 25(OH)D levels: the adjusted HRs in neurodevelopmental delay comparing children with vs without hyperinsulinemia were 1.28 (95% CI: 1.03, 1.59) for quartiles 1 (lowest), and 1.06 (95% CI: 0.78, 1.44) for quartile 4 (highest).

**Conclusions:**

Immune activation and metabolic abnormalities in fetal circulation were associated with neurodevelopmental delay in offspring, which could be attenuated by higher cord blood 25(OH)D levels in a dose-response manner.

## Introduction

Developing brain during prenatal life is more vulnerable to intrauterine adverse environment like maternal obesity, which contributes to the disruption of neurodevelopmental trajectories (1, 2). Disturbances in fetal circulation in early life may have adverse effects on long-term neurodevelopmental outcomes. Cord blood metabolic markers levels reflect fetal metabolism and the placental transfer of nutrients. However, umbilical cord metabolic markers have not generally predicted neurodevelopment in offspring.

Cord blood C-peptide, which is secreted in equimolar levels with insulin, represents the insulin-secretory activity in the fetus, and fetal hyperinsulinemia or hypoglycemia is characterized by higher levels of cord C-peptide (3). Dysfunction of insulin-secretory activity of pancreatic β-cells could induce a decrease in glucose production and cellular energy sources. Under the condition, infants were inclined to neurological impairment and later neurodevelopmental delay (4, 5). On the other hand, abundant evidence links metabolic dysfunction (such as insulin resistance and obesity) with a chronic low-grade inflammatory state characterized by the recruitment of immune inflammatory cells, abnormal cytokine, acute-phase reactant production, and inflammasome activation, a process collectively known as ‘meta-inflammation’ (6, 7).

Epidemiological and animal studies have demonstrated the detrimental impacts of maternal immune activation on altered brain structure and function in offspring (8–11). Few studies elucidated the associations between cord blood cytokines and later neurodevelopment, while the inflammatory response in the fetal circulation could be triggered by maternal inflammation (12, 13). Moreover, these studies were small-scale and have substantial variability in study design. High-sensitive C-reactive protein (hsCRP) was identified as a biomarker of systemic and low-grade chronic inflammation and was applied in clinical practice, and here cord blood hsCRP was used as a proxy for inflammatory activation in the fetal circulation (14).

Significant associations of vitamin D deficiency with metabolic complications during pregnancy including maternal obesity and gestational diabetes mellitus (GDM) (15, 16). Vitamin D supplementation may further reduce levels of meta-inflammation in obese subjects (17). In addition, vitamin D also has been implicated in the neurodevelopment of offspring and vitamin D concentrations in early life may be associated with an increased risk of neurodevelopmental disorders (18). It is currently unknown whether the association between cord blood meta-inflammatory markers and neurodevelopmental delay differ by vitamin D levels.

Thus, the prospective birth cohort study aims to evaluate the relationships between cord blood meta-inflammatory markers and neurodevelopmental delay and estimate the potential impacts of cord blood vitamin D levels on these relationships.

## Methods

### Study population

The Maternal & Infants Health in Hefei (MIH-Hefei) study is a prospective birth cohort study in three centers including Anhui Women and Child Health Care, Hospital, the First People’s Hospital of Hefei City, and the First Affiliated Hospital of Anhui Medical University. The women in the MIH-Hefei study were recruited from March 2015 to June 2021. Eligible women for the study were aged 18 to 44 years, lived in Hefei city, had no communication problems, and planned to deliver at specific participating hospitals.

Women suffered from major disorders [preexisting diabetes or hypertension (n=178), thyroid dysfunction (n=183) and heart failure (n=23)], with assisted reproductive technology (n=63) and with multiple gestations (n=206) were excluded. Moreover, newborns with birth defects (n=38), stillbirth (n=28) and/or infants without breastfeeding data (n=80) were also excluded. At the postpartum follow-up, the Denver Developmental Screening Test-II (DDST-II) and subsequent the Gesell Developmental Schedules (GDS) were used for the assessment of children’s neurodevelopmental delay aged 6-36 mon. Finally, a total of 7198 mother-infant pairs were included in the analysis (**Supplemental Figure 1**). Mothers provided written informed consent for themselves and their children before enrollment. The study was approved by the Ethics Committee of Anhui Medical University (no. 2015002).

### Cord blood metabolic biomarkers measurement

Cord blood samples were collected at delivery and were stored at -80°C until assayed. Cord metabolic markers included C-peptide, HDL-cholesterol, LDL- cholesterol, and TG. The levels of cord C-peptide were detected using an immunoassay (AutoDELFIA, PerkinElmer). Cord blood high-density lipoprotein-cholesterol (HDL-C), low-density lipoprotein-cholesterol (LDL-C), total cholesterol (TC) and triglycerides (TG) were measured by an automatic analyzer (Beckman Coulter, Brea, CA, USA). Cord blood hsCRP levels, reflecting fetal immune activation, were measured using a Beckman Coulter immunoturbidometric assay (Beckman Coulter, Brea, CA, USA). Both intra- and inter-coefficients of variation were <10%.

### Cord blood 25(OH)D measurement

Cord blood vitamin D levels (total 25(OH)D) including the concentrations of 25(OH)D_2_ and 25(OH)D_3_ in cord blood plasma, were measured using the Electrochemical Luminescence Detection Kit for Roche E601 (Sandhofer, Mannheim, Germany). Both intra- and inter-coefficients of variation were <10%.

### Assessment of neurodevelopmental delay

All the children underwent the assessment of neurodevelopmental delay using DDST-II and GDS by a specially trained examiner. The DDST-II were applied in evaluating children’s development regarding their ability to perform tasks organized in four domains: gross motor, fine motor, personal-social and language (19). Each domain was scored and evaluated as follows: pass or fail. In terms of the overall developmental assessment, children were considered as “Developmental delay” if they failed two or more domains that 75 to 90% of children of their age could pass or if they failed one or more domains that more than 90% of children younger than their age could pass. Otherwise, the development of the children was considered “Normal”.

The GDS was performed for the infants with “Developmental delay” after the assessment of DDST-II by a trained pediatrician. The GDS was designed to diagnose the neurologic and intellectual development of infants aged 4 weeks to 3 y (20). The test included 5 domains for the evaluation of the developmental quotient (DQ): adaptability (i.e., cognitive), gross motor, fine motor, language (i.e., communication), and personal-social domains (20). The mean score with SD for the overall DQ was 100 ± 15. Infants were considered as “average development” based on their scores below −1 SD from the mean score (≥ 85); “borderline development” between −1 and −2 SD from the mean score (70–85); “developmental delay” below −2 SD from the mean score (< 70). In the study, neurodevelopmental delay for infants was defined as failing more than two domains of the GDS.

### Confounding variables

Potential confounders in the study included both characteristics of mothers and infants. The information on maternal age (<30 and ≥30 years), education (≤12 and >12 years), husband’s income (<4000 and ≥4000 yuan), multipara (yes/no), and pregnancy lifestyle (the supplement of folic acid as well as iron during pregnancy and physical activity) were reported using a standardized questionnaire. Prepregnancy body mass index (BMI) (≥24 and <24 kg/m^2^) and GDM (yes and no) were obtained from medical records. At the follow-up, infants’ characteristics included mode of delivery, gestational age as well as weight at birth, prematurity status, gender and the pattern of infant feeding (exclusive breastfeeding, partial breastfeeding, and formula feeding) at 6 months *via* questionnaires.

### Statistical analyses

MIH-Hefei data by child’s sex were summarized with means (SDs) or median (interquartile range, IQR) for continuous variables and counts (frequencies) for categorical variables. Pearson’s correlation was used to evaluate the correlation between cord blood metabolic markers.

The associations of cord blood metabolic biomarkers with offspring neurodevelopmental delay were estimated utilizing Cox regression models to calculate hazard ratios (HRs). Fetal hyperinsulinemia was defined as cord blood C-peptide level above the 90th percentile (P90) in clinical practice and was used as a dichotomous variable. Cord blood hsCRP and 25(OH)D levels were used as categorical variables (by quartiles). The model adjusted several confounders including maternal age, education, husband’s income, parity, prepregnancy BMI, GDM, the supplement of folic acid as well as iron during pregnancy, physical activity, delivery mode, gestational week and the pattern of infant feeding. We further explored these relationships stratified by sex.

We examined whether the associations of cord blood C-peptide level above P90 and CRP levels (per quartile increase) varied by cord blood 25(OH)D level [used as a categorical variable (by quartiles)]. The model adjusted several confounders including maternal age, education, husband’s income, parity, prepregnancy BMI, GDM, the supplement of folic acid as well as iron during pregnancy, physical activity, delivery mode, gestational week and the pattern of infant feeding. All analyses were performed using SPSS version 22.0 software (IBM Corp).

## Results

### Participant characteristics

The primary analytic data included 7198 participants and their characteristics were summarized in **Table 1**. In the present study, 36.1% of the mothers were more than 30 y at conception and 72.9% had less than 12 years of education. 17.7% of the women were overweight/obese before conception (pre-pregnancy BMI≥24 kg/m^2^) and 18.1% of the women suffered from GDM. In terms of the infants’ characteristics, the mean (SD) gestational age and birth weight at born were 39.5 (1.4) weeks and 3403 (447.0) g, respectively. The mean (SD) cord C-peptide was 0.40(0.25) nmol/L. The median cord hsCRP and 25(OH)D level was 4.20 mg/L (IQR: 1.04-5.21) and 72.96 nmol/L (IQR: 41.17-103.94), respectively.

**Table 1:** Participant characteristics.

	Overall (n=7198)	Boys (n=3676)	Girls (n=3522)
Characteristics of pregnant women			
Age≥30 years, No. (%)	2595 (36.1)	1356 (36.9)	1239 (35.2)
Education ≤12 years, No. (%)	5249 (72.9)	2678 (72.9)	2571 (73.0)
Husband’s income < 4000 yuan, No. (%)	1407 (19.5)	694 (18.9)	713 (20.2)
Multipara, No. (%)	4417 (61.4)	2108 (59.9)	2309 (62.8)
Pre-pregnancy BMI≥24 kg/m^2^, No. (%)	1273 (17.7)	650 (17.7)	623 (17.7)
Gestational diabetes mellitus, No. (%)	1303 (18.1)	745 (20.3)	558 (15.8)
Pregnancy lifestyle			
Folic acid supplement < 1/day, No. (%)	2231 (31.0)	1144 (31.1)	1087 (30.9)
Iron supplement < 1/day, No. (%)	6575 (91.3)	3363 (91.5)	3212 (91.2)
Physical exercise < 1/day, No. (%)	5675 (78.8)	2885 (78.5)	2790 (79.2)
Infant characteristics			
Cesarean section, No. (%)	2339 (32.5)	1230 (33.5)	1109 (31.5)
Gestational week, mean (SD), weeks	39.5 (1.4)	39.5 (1.4)	39.6 (1.5)
Prematurity, No. (%)	280 (3.9)	155 (4.2)	125 (3.5)
Birthweight, mean (SD), kg	3403 (447.0)	3453 (454.4)	3352 (433.1)
Exclusive breastfeeding, No. (%)	2025 (39.2)	1103 (30.0)	922 (26.2)
Cord metabolic markers			
C-peptide, mean (SD), nmol/L	0.40 (0.25)	0.39 (0.25)	0.40 (0.25)
hsCRP, median (IQR), mg/L	4.20 (1.04-5.21)	4.23 (0.52-5.18)	4.21 (1.52-5.22)
HDL-cholesterol, mean (SD), mmol/L	0.89 (0.25)	0.86 (0.24)	0.93 (0.25)
LDL-cholesterol, mean (SD), mmol/L	0.70 (0.25)	0.68 (0.25)	0.73 (0.25)
TC, mean (SD), mmol/L	1.96 (0.64)	1.89 (0.58)	2.04 (0.69)
TG, median (IQR), mmol/L	0.36 (0.27-0.48)	0.36 (0.28-0.48)	0.35 (0.26-0.47)
25 (OH)D, median (IQR), nmol/L	72.96 (41.17-103.94)	75.82 (42.12-107.26)	70.00 (40.42-102.33)

BMI, body mass index; IQR, interquartile range.

**Supplemental Figure 2** showed summary statistics and Pearson correlation coefficients between cord blood metabolic markers. hsCRP levels were correlated to C-peptide, LDL-C, TC, TG and 25(OH)D (r: -0.16, 0.14); C-peptide levels were correlated to hsCRP, HDL-C, TG and 25(OH)D (r: -0.22, 0.10); 25(OH)D levels were correlated to other metabolic markers (r: -0.22, 0.23).

### Cord blood metabolic markers and neurodevelopmental delay

**Figure 1:**
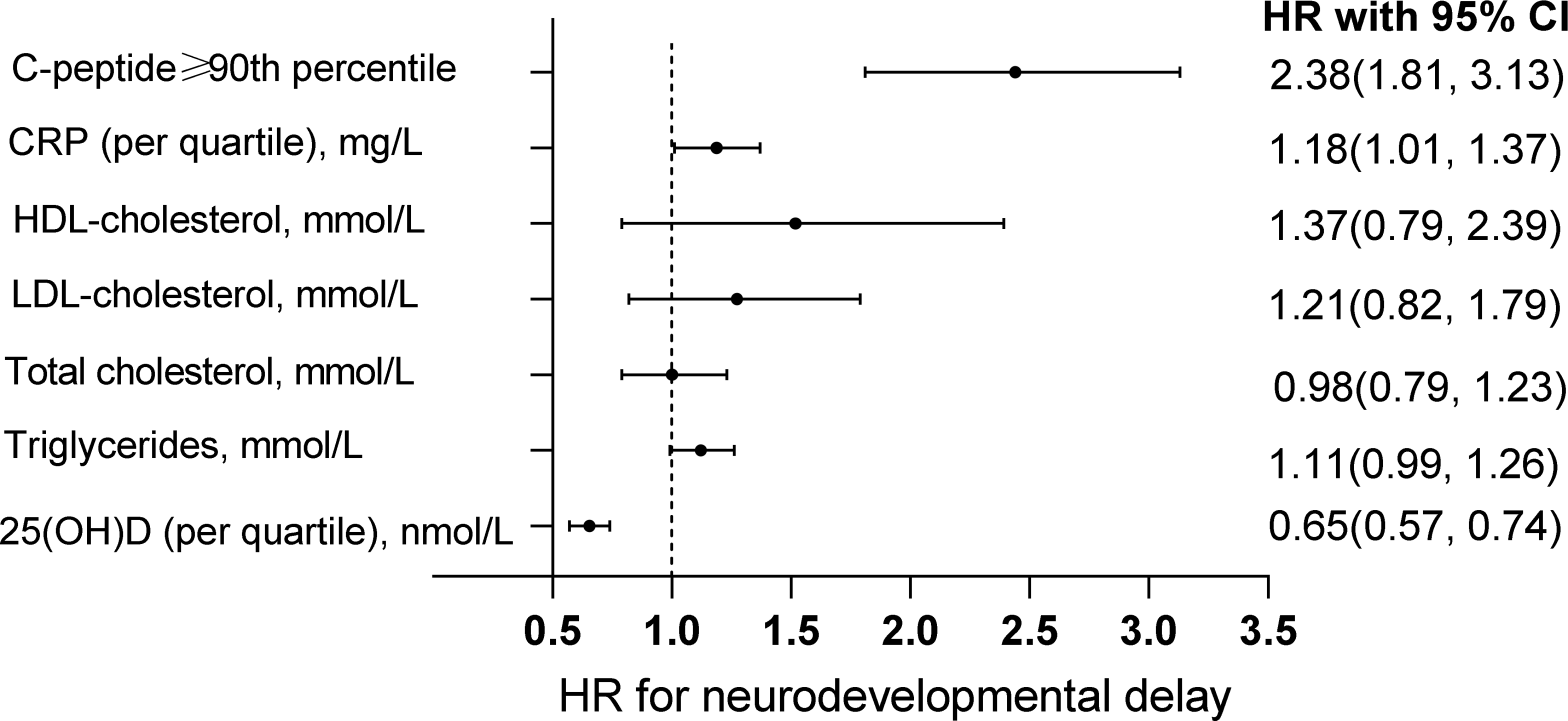
Association between cord blood markers and neurodevelopmental delay in offspring. The associations of cord blood C-peptide level above the 90th percentile, hsCRP by quartile increase, HDL-cholesterol, LDL-cholesterol, total cholesterol, triglycerides and 25(OH)D with neurodevelopmental delay in offspring. The models adjusted maternal age, education, husband’s income, parity, depressed mood, the supplement of folic acid, multivitamin as well as iron during pregnancy, physical activity, delivery mode, gestational week, sex and the pattern of infant feeding.

The associations of cord blood metabolic markers and risks of neurodevelopmental delay in offspring were exhibited in **Figure 1**. The significant associations of cord blood serum C-peptide levels above P90 with higher risks of neurodevelopmental delay were observed [HR with 95% CI: 2.38(1.81, 3.13)]. Similarly, our analysis demonstrated the significant relationship between cord hsCRP levels by quartile increase and neurodevelopmental delay in offspring [HR with 95% CI: 1.18(1.01, 1.37)]. In addition, cord 25(OH)D levels by quartile increase were associated with decreased risk of neurodevelopmental delay [HR with 95% CI: 0.65(0.57, 0.74)]. However, these significant associations with other cord blood metabolic markers were not observed.

### Sex-specific effects

We further explored the sex-specific effects on the relationships between cord blood metabolic markers and neurodevelopmental delay. The stratified analysis indicated that cord hsCRP levels by quartile increase were associated with higher risks of neurodevelopmental delay in boys [HR with 95% CI: 1.29(1.02, 1.63)] but not girls (**Table 2**). In addition, compared with girls, boys exposed to high levels of cord blood TG have a higher neurodevelopmental delay risk [HR with 95% CI: 1.17(1.03, 1.33)].

**Table 2:** Sex-specific effects on the associations of cord blood markers with neurodevelopmental delay.

Cord metabolic markers	Boys (n=3676)		Girls (n=3522)	
	Hazard Ratio (95% CI)	*P* Value	Hazard Ratio (95% CI)	*P* Value
C-peptide≥90th percentile	2.20 (1.49, 3.26)	<0.001	2.63 (1.79, 3.86)	<0.001
CRP (per quartile), mg/L	1.29 (1.02, 1.63)	0.031	1.10 (0.89, 1.34)	0.381
HDL-cholesterol, mmol/L	1.56 (0.92, 2.65)	0.298	1.21 (0.80, 1.82)	0.321
LDL-cholesterol, mmol/L	1.36 (0.78, 2.37)	0.523	1.12 (0.80, 1.57)	0.380
TC, mmol/L	1.13 (0.83, 1.53)	0.435	0.86 (0.61, 1.22)	0.393
TG, mmol/L	1.17 (1.03, 1.33)	0.019	0.94 (0.64, 1.38)	0.746
25 (OH)D (per quartile), nmol/L	0.62 (0.52, 0.74)	<0.001	0.68 (0.57, 0.82)	<0.001

The model was used to determine the associations of cord blood markers with neurodevelopmental delay, adjusting maternal age, education, husband’s income, parity, depressed mood, the supplement of folic acid, multivitamins as well as iron during pregnancy, physical activity, delivery mode, birth weight, prematurity, gestational week and the pattern of infant feeding.

### Cord blood metabolic markers and neurodevelopmental delay by 25(OH)D levels

**Figure 2:**
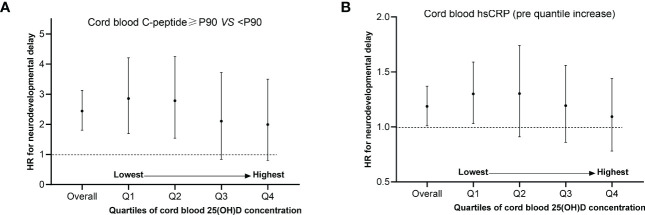
Potential relationships between C-peptide levels above P90, cord hsCRP levels by quartile increase and neurodevelopmental delay risk stratified by level of cord blood 25(OH)D concentration. **(A, B)** Adjusted maternal age, education, husband’s income, parity, depressed mood, the supplement of folic acid, multivitamin as well as iron during pregnancy, physical activity, delivery mode, gestational week, sex and the pattern of infant feeding. The no. of quartile 1 (n=1809), quartile 2 (n=1882), quartile 3 (n=1742) and quartile 4 (n=1763) for cord blood 25(OH)D concentration was analyzed.

Potential relationships between cord hsCRP levels by quartile increase, C-peptide levels above P90 and neurodevelopmental delay risk were performed in a dose-dependent manner by cord blood 25(OH)D concentration (**Figure 2**). Our results showed that by quartiles of cord blood 25(OH)D, the adjusted HRs in neurodevelopmental delay comparing children with vs without hyperinsulinemia were 1.28 (95% CI: 1.03, 1.59) for quartiles 1 (lowest), 1.26 (95% CI: 0.91, 1.74) for quartile 2, 1.16 (95% CI: 0.86, 1.56) for quartile 3, and 1.06 (95% CI: 0.78, 1.44) for quartile 4 (highest). Similarly, the adjusted HRs in neurodevelopmental delay comparing children with vs without higher levels inflammation (per quartile increase in CRP) were 2.67 (95% CI: 1.69, 4.21) for quartiles 1 (lowest), 2.56 (95% CI: 1.54, 4.25) for quartile 2, 1.76 (95% CI: 0.84, 3.72) for quartile 3, and 1.63 (95% CI: 0.48, 5.59) for quartile 4 (highest). Specifically, sex-stratified cord hsCRP associations were stronger among boys (**Supplemental Figures 2A, B**).

## Discussion

In the prospective birth cohort study, after adjusting a series of confounders, we found that offspring exposed to higher levels of cord blood serum C-peptide level above P90 or cord blood hsCRP had an increased risk of neurodevelopmental delay. This risk could be attenuated by higher cord blood 25(OH)D levels. Overall, our results suggested that meta-inflammatory markers in the fetal circulation could be implicated in later neurodevelopmental delay and addressed the potentially protective impacts of vitamin D levels.

The current analysis reported the significance of cord serum C-peptide levels above P90 (defined as fetal hyperinsulinemia) to increased risks of neurodevelopmental delay. Increasing evidence has indicated that cord blood C-peptide was associated with maternal insulin sensitivity, fetal hyperinsulinemia and/or neonatal hypoglycemia (21, 22). There have been few studies to date on the impact of cord serum C-peptide on neurodevelopmental delay. Moreover, possible biological mechanisms linking cord blood C-peptide with brain development remain elusive. Alternatively, cord blood C-peptide could cross the placental barrier, resulting in dysregulated, uncoupled glucose and fuel metabolism and a subsequent decrease in glucose production. Under these circumstances, fetuses and infants are disposed to brain injury or later neurological impairment. Another possible explanation was that the higher levels of C-peptide induced the upregulation of nitric oxide synthase underlying the nitric oxide signaling pathway (23). Excessive nitric oxide was detrimental to cognitive dysfunction and neurological changes, especially for fetal and infant brain development (24). Thus, the present study provided new data that higher cord C-peptide was associated with neurodevelopment in offspring. Our results suggested that metabolic disorders in maternal-fetal circulation could be implicated in the neurodevelopment of offspring.

On the other hand, increasing evidence has demonstrated the relationships between maternal immune activation and altered brain development in neonates and toddlers (10, 11). Recently, data from the Generation R study found significant associations of maternal continuous CRP levels with lower cerebellar volume in late childhood (25). Few studies explored the associations between cord blood cytokines and later neurodevelopment, while the inflammatory response in the fetal circulation could be triggered by maternal inflammation (12, 13). A longitudinal study inborn small-for-gestational-age and preterm birth found that higher levels of cord blood tumor necrosis factor-α (TNF-α) were associated with a decrease in verbal intelligence quotients (13). Conversely, another nested case-control analysis indicated no significant relationship between cord serum inflammatory cytokine levels (like TNF-α and interleukin 8) and neurodevelopmental delay in children (12). These studies were small-scale and the conclusion from the retrospective studies remained inconsistent. In the present prospective cohort study, cord blood hsCRP used as a proxy for fetal inflammatory activation was associated with increased risks of neurodevelopmental delay in children. Mechanistically, microglia play a critical role in regulating neuronal differentiation and neural circuit formation during the developing brain (26–29). Activated microglia by fetal immune activation induces the release of pro-inflammatory cytokines, resulting in neurodevelopmental delays in children. Therefore, our results suggested a positive association between fetal neuroinflammation and poorer neurodevelopment. Considering the interactions of immune activation in the maternal-fetal circulation, maternal inflammation was likely to be a potential intervention target for the prevention of abnormal neurodevelopment and adhering to the higher anti-inflammatory potential of maternal diet pattern was associated with lower risks of neurodevelopmental abnormalities (30).

The potential effects of vitamin D on immune function and cellular metabolic pathways have been recognized (31). The activated form of Vitamin D (1,25(OH)_2_D_3_) or its analogs bind to vitamin D receptor (VDR) and further induce VDR physiological functions (32). Macrophage VDR activation might inhibit inhibitor of kappa β kinase (IKKβ)-induced inflammation activity *via* suppressing nuclear factor-κB (NF-κB) signaling activation (33). NF-κB signaling plays a pivotal role in inflammatory responses and energy homeostasis metabolic diseases such as obesity and type 2 diabetes (34). Anti-inflammatory inhibition of NF-κB signaling by VDR activation improves insulin resistance in obese mice (35). A meta-analysis including five randomized controlled trials (RCT) involving 310 women found that GDM women with vitamin D supplement may lead to an improvement in serum metabolic a (36)nd inflammatory markers such as TC and hs-CRP (36). In addition, vitamin D is directly involved in the physiological process of the developing brain such as neurotransmitter synthesis and calcium (37). Results from a register-based cohort study showed that early life vitamin D status was associated with autism spectrum disorders (18). However, no study reported that early life vitamin D could modify the relationships between cord blood meta-inflammation and neurodevelopment. Hence, our results suggested that early life vitamin D exposure to fetal immune responses and metabolic disorders might represent a plausible mechanism linking early life meta-inflammation to neurodevelopmental delay in humans.

In line with the literature, we found the sex-specific effect of fetal immune activation on later neurodevelopment in males. Growing evidence has demonstrated that male offspring may be more vulnerable to maternal metabolic disorders and immune activation during pregnancy, which could lead to later poorer brain development (38–40). Our results provided new evidence on the sex-specific effect on the relationship between intrauterine exposure to meta-inflammation and later neurodevelopmental delay.

The current analysis has some strengths. First, it is the first time to investigate the impacts of cord meta-inflammatory markers on neurodevelopmental outcomes in a large-scale prospective birth cohort study. Our study has demonstrated that the associations of cord C-peptide and hs-CRP with neurodevelopmental delay may be attenuated by higher levels of vitamin D. Third, the current analysis included a series of potential confounders related to mothers and infants such as characteristics of pregnancy lifestyle. However, the present study also has several limitations. First, information on maternal diet during pregnancy was not detailed in our study. Gestational diet has been demonstrated to be related to both cord blood metabolic biomarkers and offspring neurodevelopment (41, 42). Second, a single marker for fetal immune activation (cord blood hsCRP) is one of the current analyses, while hsCRP was identified as a biomarker of systemic and low-grade chronic inflammation, and was applied in clinical practice. Data on the measurements of multiple inflammatory cytokines were not available in the study. In this large-scale prospective cohort study, several conventional cord metabolic markers (such as C-peptide, and high-density lipoprotein-cholesterol) were measured due to the limited research funding. More inflammatory measures and metabolomics in the future studies would add in future studies. Third, although the positive relationships between cord meta-inflammatory markers and neurodevelopmental delay in offspring were observed, the assessment of subsequent neurodevelopmental trajectories in childhood and even adolescence were required.

## Conclusion

In the prospective birth cohort study, we found that the higher levels of cord blood C-peptide and hsCRP were associated with increased risks of neurodevelopmental delay, which might be modified by adequate cord blood 25(OH)D levels. Our results suggest that metabolic disorders and immune activation in fetal circulation may adversely affect the programming of the brain development in offspring and optimal vitamin D levels could prevent them from the later neurodevelopmental delay. Further clinical trials on the effect of vitamin D supplementation during pregnancy on neurodevelopmental outcomes are necessary to confirm this benefit.

## Data availability statement

The original contributions presented in the study are included in the article/**Supplementary Material**. Further inquiries can be directed to the corresponding authors.

## Ethics statement

The studies involving human participants were reviewed and approved by the Ethics Committee of Anhui Medical University. The patients/participants provided their written informed consent to participate in this study.

## Author contributions

PW designed the study, interpreted the data and wrote the manuscript. PW, LW and W-JY conducted data analysis and wrote the manuscript. R-XT and YZ advised on statistical methods and participated in the acquisition of the data. W-JY, P-PL and Z-YS designed the study, and interpreted the data, X-MJ and PZ were the guarantors of this work and, as such, had full access to all the data in the study and take responsibility for the integrity of the data and the accuracy of the data analysis. All authors approved the final version of the manuscript. All authors contributed to the article and approved the submitted version.
