# Supplementary material for: Associations of cord blood meta-inflammation and vitamin D with neurodevelopmental delay: A prospective birth cohort study in China
Source: Front Immunol. 2023 Jan 4;13:1078340. doi: 10.3389/fimmu.2022.1078340 (PMC9846620; doi:10.3389/fimmu.2022.1078340)
Supplement: Supplementary file 1 [file DataSheet_1.docx]

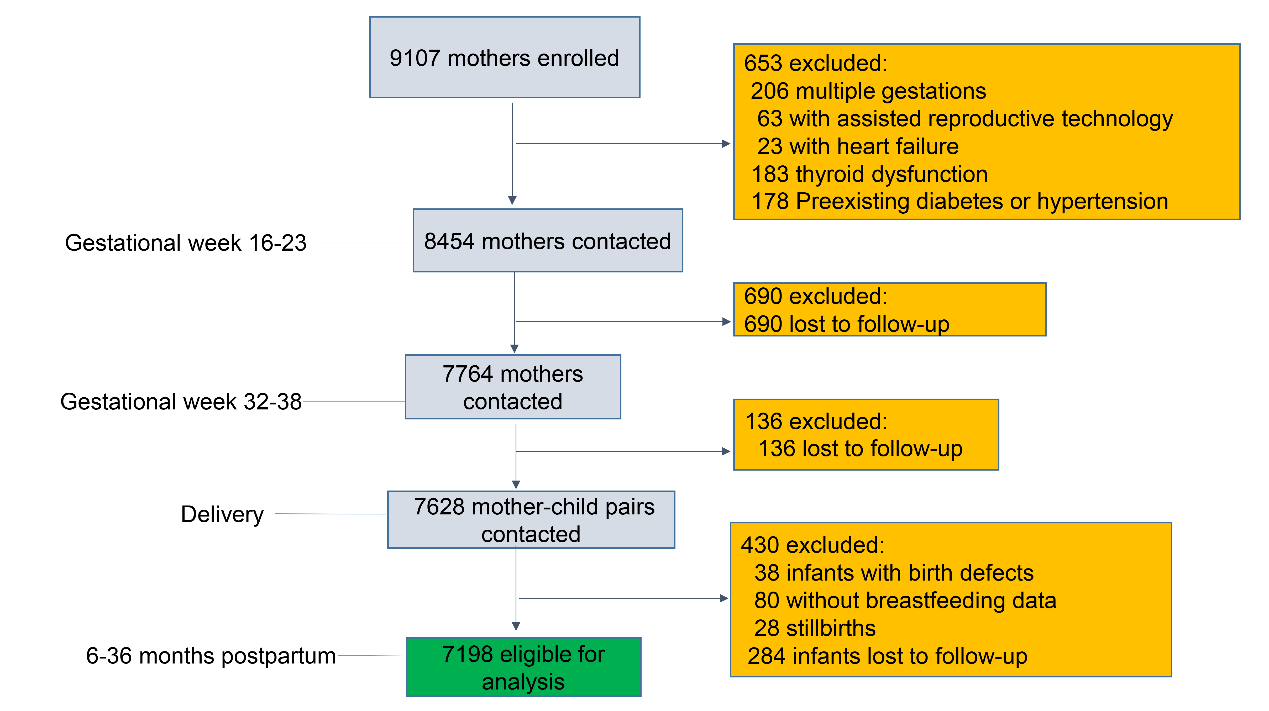


Supplemental Figure 1. Flowchart illustrating enrolment.


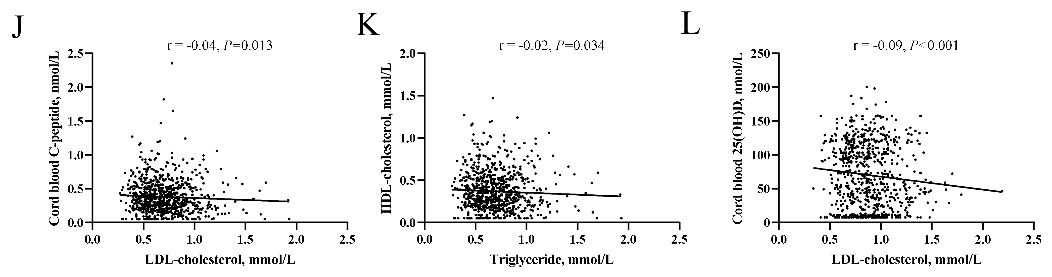

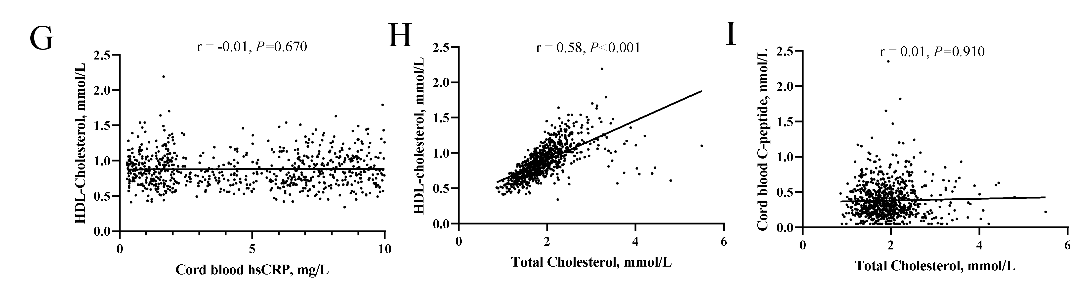

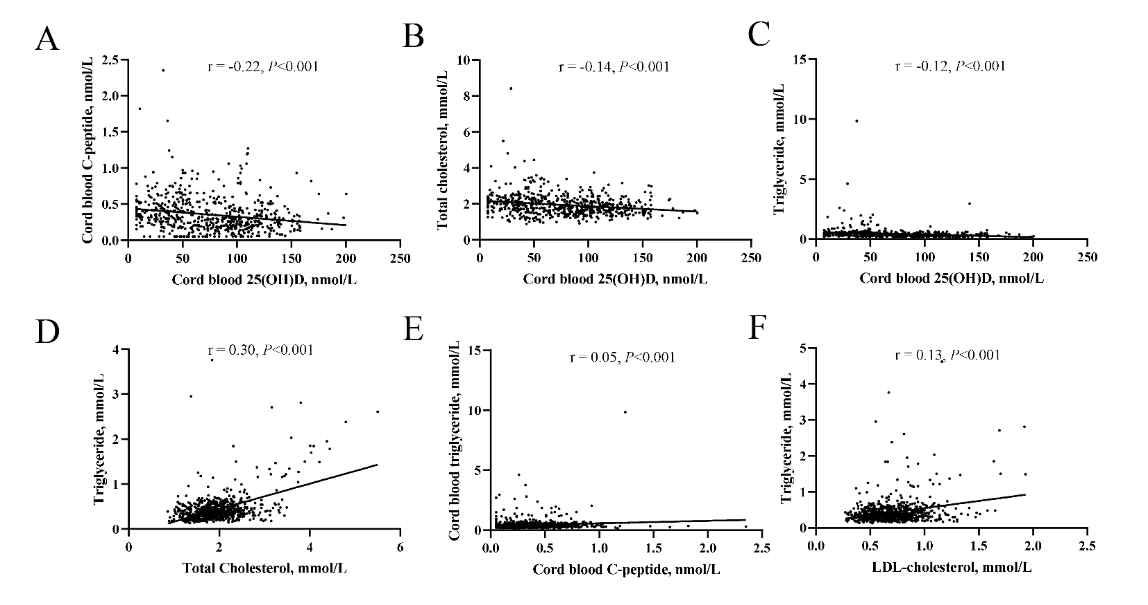


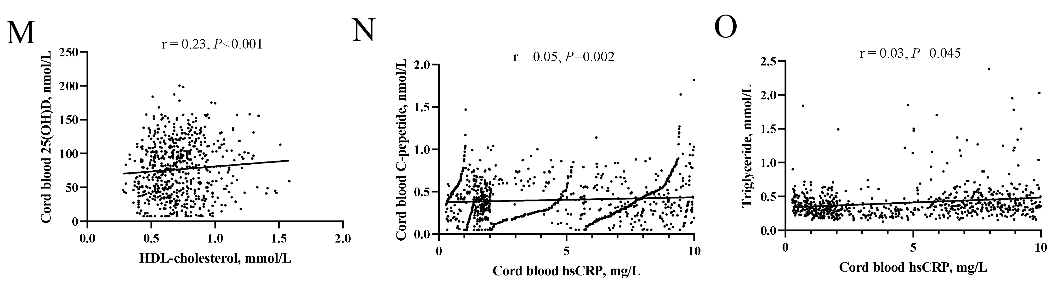

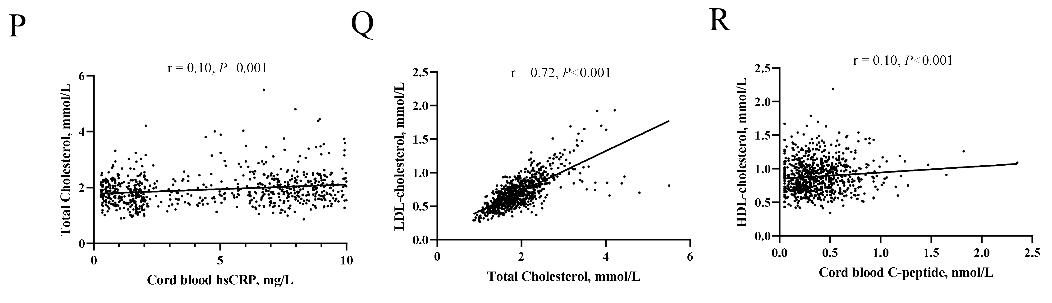

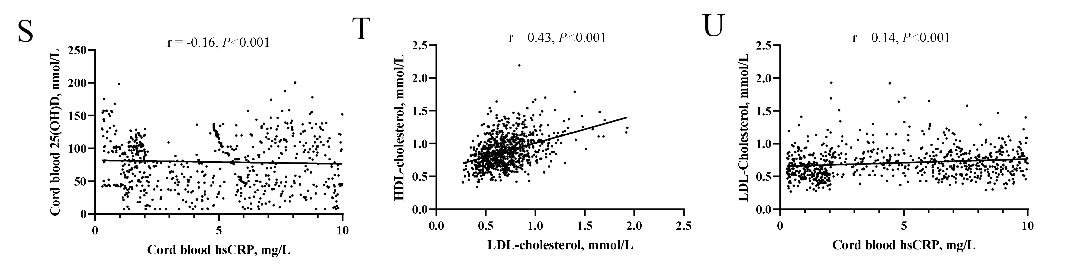


Supplemental Figure 2. Pearson correlation coefficients between cord blood metabolic markers.


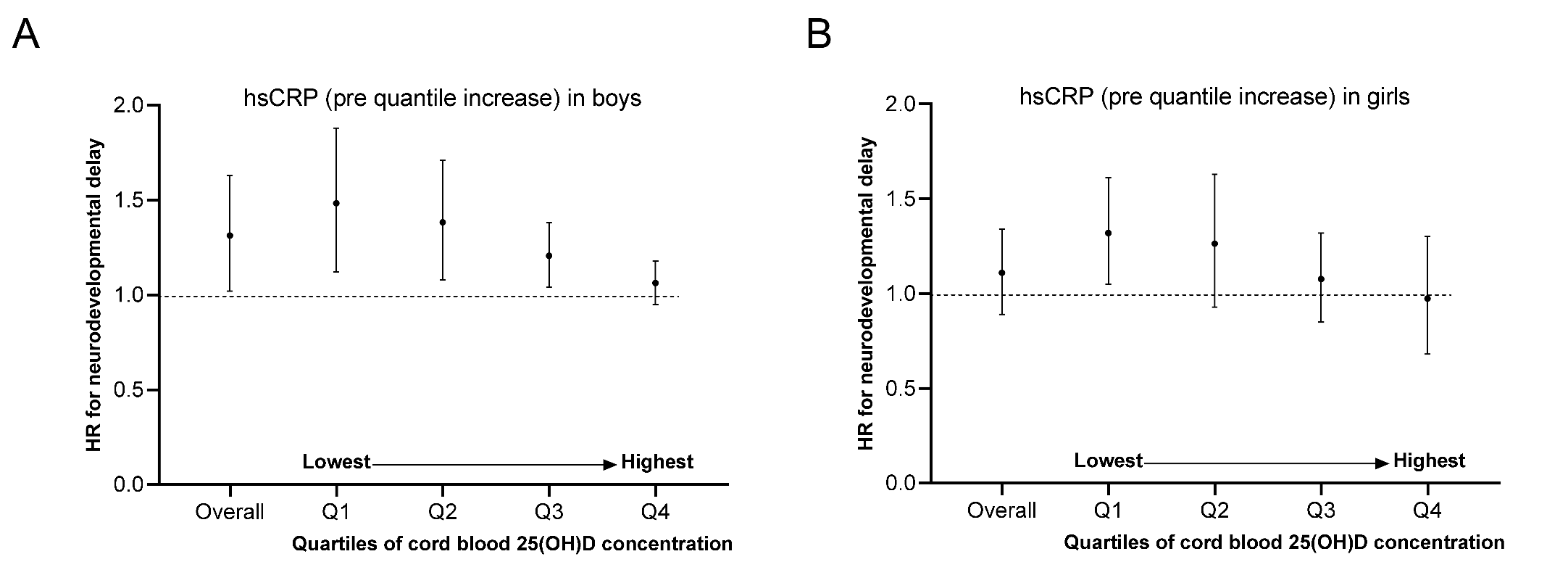
Supplemental Figure 3. Association between cord blood hsCRP (per quartile increase) and neurodevelopmental delay in offspring stratified by sex. A) and B) adjusted maternal age, education, husband’s income, parity, depressed mood, the supplement of folic acid, multivitamin as well as iron during pregnancy, physical activity, delivery mode, gestational week, sex and the pattern of infant feeding. A) The no. of quartile 1 (n=837), quartile 2 (n=830), quartile 3 (n=943) and quartile 4 (n=1066) for cord blood 25(OH)D concentration was analyzed. B) The no. of quartile 1 (n=972), quartile 2 (n=1052), quartile 3 (n=800) and quartile 4 (n=698) for cord blood 25(OH)D concentration was analyzed.
